# miR-126-5p protects from URSA via inhibiting Caspase-1-dependent pyroptosis of trophoblast cells

**DOI:** 10.1007/s00018-025-05713-w

**Published:** 2025-05-15

**Authors:** Xiaoxiao Zhu, Ke Xu, Shuang Ai, Yingjie Zhang, Chu Chu, Ran Wei, Shufeng Gao, Lu Liu, Wei Li, Yunhong Zhang, Siambi Kikete, Xinkui Liu, Zhen Zhang, Xia Li

**Affiliations:** 1 Innovative Institute of Chinese Medicine and Pharmacy, Shandong University of Traditional Chinese Medicine, 4655 Daxue Road, Changqing District, Jinan, 250399 China; 2 Key Laboratory of Traditional Chinese Medicine Classical Theory, Ministry of Education, Shandong University of Traditional Chinese Medicine, Jinan, People’s Republic of China; 3 Affiliated Hospital of Shandong University of Traditional Chinese Medicine, Jinan, China; 4 The First Clinical College of Medicine, Shandong University of Traditional Chinese Medicine, Jinan, China; 5 School of Clinical and Basic Medical Sciences, Shandong First Medical University & Shandong Academy of Medical Sciences, Jinan, China; 6 School of Health Sciences, Department of Pharmacognosy and Pharmaceutical Chemistry, Kenyatta University, Nairobi, 00609 Kenya; 7 College of Traditional Chinese Medicine, Shandong University of Traditional Chinese Medicine, Jinan, China

**Keywords:** Unexplained recurrent spontaneous abortion, Pyroptosis, miR-126-5p, Caspase-1, Trophoblast

## Abstract

Unexplained recurrent spontaneous abortion (URSA) is a distressing pregnancy complication that seriously threat to women's reproductive health. Trophoblast pyroptosis was involved in the occurrence of URSA, but the potential mechanism remains unclear. In this work, we found *CASP1* transcription and the level of pyroptosis were significantly elevated in the villous tissues of URSA patients. Suppression of cell pyroptosis by Gasdermin-D (GSDMD) or Caspase-1 inhibitors can reduce embryo resorption rate of URSA mice, while Caspase-1 over-expression in normal pregnant (NP) mice can aggravate embryo resorption. Meanwhile, a pronounced decline in the expression of microRNA-126-5p (miR-126-5p) was found in URSA patients, which was inversely related to *CASP1* expression. Over-expression of miR-126-5p restrained trophoblast pyroptosis via inhibiting Caspase-1/GSDMD signaling pathway by direct binding to 3’-UTR of *CASP1*. Moreover, experiments in vivo substantiated that up-regulation of miR-126-5p effectively suppressed Caspase-1-mediated pyroptosis in placental tissue and significantly reduced embryo resorption rate. Collectively, these results underscored that diminished miR-126-5p expression plays a crucial role in URSA by enhancing trophoblast pyroptosis through activating Caspase-1/GSDMD signaling pathway. As a result, miR-126-5p shows significant promise as a possible biomarker for diagnosis and treatment of URSA.

**Supplementary Information:**

The online version contains supplementary material available at 10.1007/s00018-025-05713-w.

## Introduction

Recurrent spontaneous abortion (RSA) is characterized by two or more uninterrupted spontaneous pregnancy losses with the same partner before 20–24 weeks of gestation, which affect about 1%- 5% of pregnant women [1, 2]. The pathogenic factors of RSA are complex. In addition to established causes including genetic, endocrinologic, anatomic and infectious factors, approximately half of RSA cases have unknown etiology and are classified as unexplained recurrent spontaneous abortion (URSA) [3, 4]. The treatment of URSA primarily involves progesterone supplementation, anticoagulation therapy, and immunotherapy. However, the clinical efficacy of these interventions remains constrained by their non-specific mechanisms and lack of individualized therapeutic strategies. Therefore, further in-depth research into the pathogenic mechanisms of URSA is essential to develop more effective and personalized therapeutic interventions. Normal pregnancy, similar to successful allogeneic transplantation, depends on the successful establishment and retention of maternal fetal immunotolerance for the embryo to grow and develop until delivery [5]. Imbalance of immunotolerance and inflammation at the maternal fetal interface can lead to URSA [6, 7]. The trophoblasts are the unique embryo sourced cells that directly communicate with decidual immune cells, and the interaction is essential in the foundation of immunologic tolerance [8, 9]. The malfunction of human trophoblast cells result in the imbalance of immunological tolerance, which is critical in the contribution of URSA occurrence [10].

Pyroptosis is a type of programmed cell death primarily driven by Gasdermin-D (GSDMD) protein. Pyroptosis can be categorized into two primary pathways based on the mechanism of activation: classical and non-classical pathway [11, 12]. As for classical pathway, activation of Caspase-1 initiates cleavage of GSDMD, leading to generation of a toxic GSDMD N-terminal fragment (GSDMD-N), which can drill holes on cytomembrane. Meanwhile, activated Caspase-1 can cleave the immature IL-1β and IL-18, which are exported to the extracellular space through cytomembrane holes, and thereby promoting immune cell aggregation and triggering robust inflammatory responses [13–16]. Pyroptosis is emerging as a key defense strategy for hosts against pathogens in normal physiological conditions, however, excessive pyroptosis may cause prolonged and sustained inflammatory reactions [17]. Recently, pyroptosis has been reported to participate in the pathogenesis of inflammatory diseases, including live diseases, nervous system diseases, and cardiovascular diseases [18–22]. However, limited investigation has been conducted to reveal the underlying roles of pyroptosis in URSA.

MicroRNAs are small in length with approximately 22 nucleotides, which function as negative gene transcriptional regulators by binding to the 3'UTR of the target genes [23, 24]. MiRNAs were involved in multiple physiological conditions, including cell differentiation, apoptosis, angiogenesis, as well as disease development [25, 26]. Recently, miRNA has been reported to be involved in URSA by affecting the migration, and invasion of trophoblast [27, 28]. Besides, it also proved that miRNAs function through pyroptosis to contribute to various diseases such as sepsis-related acute lung injury and diabetes [29, 30]. However, the regulatory impact of miRNAs on the trophoblasts pyroptosis in URSA still need to be elucidated.

Here, we aimed to investigate the association between trophoblast cell pyroptosis and URSA, as well as to explore the regulatory roles of miRNAs in these processes. We observed a significant up-regulation of Caspase-1 and Caspase-1 mediated cell pyroptosis in villous tissues of URSA patients. Inhibiting Caspase-1 and cell pyroptosis reduced pyroptosis levels in placental tissue and mitigated embryo absorption in URSA mice. Furthermore, in villous tissues of URSA patients, miR-126-5p was down-regulated, which negatively correlated with CASP1 mRNA levels. Mechanistically, miR-126-5p directly suppressed trophoblast cell pyroptosis through the Caspase-1/GSDMD signaling pathway by targeting the 3'UTR of CASP1. Decreased miR-126-5p relieved inhibition on Caspase-1, thereby promoting trophoblast cell pyroptosis and contributing to URSA development. These findings provide potential therapeutic strategies for URSA treatment.

## Materials and methods

### Patients

All participants (35 URSA and 35 NP) came from the Department of Obstetrics and Gynecology at Affiliated Hospital of Shandong University of Traditional Chinese Medicine (TCM) between August 2022 and August 2023 and signed informed consent forms. NP participants were required to:1. Have no history of adverse pregnancy outcomes (stillbirth, fetal demise, ectopic pregnancy, or spontaneous miscarriage), 2. Show no current threatened abortion symptoms, 3. Exhibit confirmed fetal cardiac activity, 4. Have no parental/embryonic chromosomal abnormalities. URSA patients were required to: 1. Have a history of at least two continuous spontaneous early abortions ranging from 6 to 10 weeks of gestation, 2. Confirm miscarriage through blood tests and ultrasound evaluations, with diagnostic criteria including the absence of fetal cardiac activity on ultrasound or a significant decrease in hCG levels, 3. Exclude individuals with infections, metabolic or endocrine conditions, autoimmune disorders, or chromosomal abnormalities originating. The study received approval from the Ethics Committee of Affiliated Hospital of Shandong University of Traditional Chinese Medicine (TCM) (Ethical number: (2022) Lun Shen No. (008)-KY). Participant details were provided in Table 1.

**Table 1:** Clinical characteristics of patients

Patients	URSA (mean ± SEM, n = 35)	NP (mean ± SEM, n = 35)	*P*
Age (years)	33.46 ± 0.90	31.09 ± 0.98	0.0828
Number of miscarriages	2.63 ± 0.11	0	< 0.0001
Gestation age(weeks)	7.49 ± 0.21	7.66 ± 0.26	0.61

### Sample

Venous blood of URSA patients after abortion and NP women before abortion were collected by conventional blood collection tube (Becton Dickinson). The serum was collected after ultra-centrifugation and stored at −80℃. Villous tissues were obtained from patients using the negative pressure method, followed by fixation in 4% paraformaldehyde, or quick freezing in liquid nitrogen.

### Cell culture

293 T, HTR-8/SVneo, JEG-3 cells were derived from repository at the Chinese Academy of Sciences. Cells were cultured in 1640 medium (Bioind, Kibbuiz, Israel) in a 37 °C full humidity cell culture chamber containing 5% CO2. To investigate the effect of miR-126-5p on pyroptosis of trophoblast cells, miR-126-5p mimics, inhibitor, mimics control (NC), inhibitor NC (INC) were transfected into HTR-8/SVneo and JEG-3 cells at a concentration of 100 nM for 24 h, 40 mmol/L Homocysteine (Hcy) was added to induce cell pyroptosis.

### 4D label free quantitative proteomics

Villous tissues from URSA patients and NP women (n = 3) were collected for 4D label free quantitative proteomics. The work was performed by Jingjie Biotechnology Co., Ltd (Hangzhou, China). Briefly, six samples were ground into powder using liquid nitrogen, and then subjected to ultrasonic lysis by adding lysis buffer. After passing the quality inspection, an equal amount of sample protein is taken for enzymatic hydrolysis. To screen for differential proteins, fold change (URSA/NP) > 1.2 and <  − 1.2, *P* < 0.05 was used.

### RNA-sequencing (RNA-seq)

Villous tissues of URSA patients and NP women (n = 3) were collected for RNA-seq by OE Biotech (Shanghai, China). The experimental procedure included total RNA extraction, RNA quality testing, removal of rRNA followed by RNA fragmentation, then reverse transcription to generate cDNA, end-complementation, addition of A-tails, addition of splices followed by PCR amplification, and then onboard detection on the Illumina platform. Genes that fold change (URSA/NP) > 1.5, *P* < 0.05 were used for GO enrichment.

### qRT-PCR

The total RNA of human villous and mouse placenta were extracted by TRIzol reagent (Invitrogen, Waltham) and the total RNA of cells was extracted through SPARKeasy Cell RNA Kit (SparkJade, Shandong, China). The initial step involved the reverse transcription of miRNA and mRNA, utilizing the miRNA cDNA synthesis kit from Vazyme (Nanjing, China) and the PrimeScript RT kit provided by Toyobo (Osaka, Japan), respectively. qRT-PCR was performed on a Quantstudio 1Plus instrument (Thermo Fisher, USA) using SYBR Green (CW2601, cwbio, China). The primer sequences were showed in Table 2.

**Table 2:** Primers used in the study

Gene	Forward primer (5'−3')	Reverse primer (5'−3')
Homo sapiens		
*CASP1*	GTGCAGGACAACCCAGCTAT	TGCGGCTTGACTTGTCCATT
*ACTB*	CATGTACGTTGCTATCCAGGC	CTCCTTAATGTCACGCACGAT
Mus musculus		
*Casp1*	CGTACACGTCTTGCCCTCAT	AACTTGAGCTCCAACCCTCG
*Actb*	GGCTGTATTCCCCTCCATCG	CCAGTTGGTAACAATGCCATGT
miRNAs		
miR-126-5p	TGGTGGAGGCATTATTACTTTTGG	GTGCAGGGTCCGAGGT
miR-181 d-5p	CTCATAAACATTCATTGTTGTCGG	TATCGTTGTTCTCCACTCCTTCAC
miR-181c-5p	AGCAACATTCAACCTGTCGG	AGAGCAGGGTCCGAGGTA
U6	CGCTTCGGCAGCACATATAC	TTCACGAATTTGCGTGTCATC

### Western blotting analysis (WB)

The protein of human villous tissue, mouse placental tissue and cells were extracted with RIPA lysis buffer. Primary antibodies were used for protein blotting including Caspase-1 (1:1000, ab1872, Abcam, UK), GSDMD (1:1000, NBP2-33,422, Novus biological, USA) and GAPDH (1:10,000, ab181603, Abcam, UK).

### Immunofluorescence (IF)

The villous tissue was preserved using 4% paraformaldehyde, subsequently embedded in paraffin, and cutted into 5 μM paraffin sections. Following the processes of dewaxing, hydration, and antigen retrieval, the sections were treated with goat serum for 30 min before being incubated with antibody specific for Caspase-1 (22,915–1-AP, Proteintech, Chicago, USA) or GSDMD (NBP2-33,422, Novus, Colorado, USA) overnight at 4 °C. Finally, the nucleus was stained by 6-diamino-2-phenylindole (DAPI) and fluorescence microscopy (IX73; Olympus, Japan) was used for imaging and analysis.

Cells were preserved using 4% paraformaldehyde for 30 min, and an 8 min treatment permeabilized via 0.3% Triton X-100. Subsequently, a blocking step with 10% normal goat serum was performed for 1. The primary antibody were incubated overnight at 4 °C, and then, secondary antibody FITC goat anti-rabbit IgG (ZSBIO, Beijing, China) was incubated in the dark at room temperature for 1.5 h. The nuclei were stained using DAPI and analyzed by fluorescence microscopy (BZ-X800LE, Keyence, Japan).

### Fluorescence in situ hybridization (FISH)

The localization and expression of miR-126-5p in villous tissue from patients with NP and URSA were detected though FISH. The preparation of villous tissue slices was the same as for immunofluorescence preparation. The slices were incubated at 37 °C with a diluted solution of proteinase K for 20 min. After dehydration with gradient alcohols, the slices were incubated with a denaturing agent in a 78 °C oven for 8 min. Next, the slices were incubated with Cy3-marked miR-126-5p probe (Cy3-5'-CGCGTACACAAAAGTAATAATG-3') in a dark environment. The nucleus was stained by DAPI, and the image was obtained by fluorescence microscopy (Revolve, Echo, USA).

### Microscopic observation of cell pyroptosis

To observe the morphology of trophoblast pyroptosis, HTR-8/SVneo cells were inoculated in 24-well plates (Corning, Shanghai, China) and treated with miR-126-5p mimics/NC, miR-126-5p inhibitor/INC for 24 h, 40 mmol/L Homocysteine (Hcy) was added to induce cell pyroptosis as described previously [31]. After 8 h the dynamic process of cell pyroptosis was continuously filmed with celldiscovery7 (ZEISS, Aalen, Germany) while capturing static cell images.

### Dual-luciferase reporter assay

The presence of complementary binding sites between the Caspase-1 mRNA 3'UTR and miR-126-5p was predicted by TargetScan (https://www.targetscan.org/vert_72) and a luciferase reporter gene assay was performed to determine the combination. Fragments of wild-type (WT) and mutant (MUT) human Caspase-1 mRNA 3'UTR were amplified and inserted into the pGL3-3 M Luc vector (Promega, Madison, WI, USA) to generate luciferase reporter vectors. 293 T cells were co-transfected with WT or MUT luciferase reporter plasmid and miR-126-5p mimics or NC with a final concentration of 100 nM. After 24 h, cells were collected with GloMax 20/20 luminometer (Promega, Madison, WI, USA) under recommended conditions for application in dual luciferase reporter gene assay system (Promega, Madison, WI, USA). The ratio of firefly luciferase luminescence to that of sea kidney luciferase was calculated.

### Animal experiments

After one week of adaptive feeding, the URSA(CBA/J females × DBA/2 males) and NP (CBA/J females × BALB/c males) model mice were constructed as described previously[32]. Designate the day when the vaginal plug appears as the 0.5th day of pregnancy. The animal experimental protocols were adhered to the guiding principles established by the Experimental Animal Management Committee of Shandong University of TCM. Great emphasis was placed on reducing the number of experimental animals and minimizing their distress during the experiments to the greatest extent feasible.

NP mice were divided into three groups by the stochastic method: plasmids vector group (treatment with plasmids), Flag mCasp1 group (treatment with Caspase-1 over-expression plasmids), Flag mCasp1 + disulfiram (DSF, S1680, Selleck Chemicals, Houston TX) group, (treatment with Caspase-1 over-expression plasmids and pyroptosis inhibitor DSF). URSA mice were divided into nine groups by the stochastic method: URSA group, DSF group (treatment with DSF), Belnacasan (VX-765, S2228, Selleck Chemicals, Houston TX) group (injected VX-765), miR-126-5p mimics group (injected miR-126-5p mimics), NC group (injected mimics NC), miR-126-5p mimics + Flag-mCasp1 group (injected miR-126-5p mimics and Flag-mCasp1), INC group (injected INC), miR-126-5p inhibitor group (injected miR-126-5p inhibitor), miR-126-5p inhibitor + VX-765 group (injected miR-126-5p inhibitor and VX-765). Mice receives treatment with plasmids vector, Caspase-1 over-expression plasmids, NC/miR-126-5p mimics and INC/miR-126-5p inhibitor through tail vein injection method using an in vivo transfection reagent (18,668–11-1, Entranster™-in vivo, Engreen Biosystem Co,Ltd, China). The plasmids vector and Flag-mCasp were administered at a dose of 12.5 µg/kg, while the NC/miR-126-5p mimics and INC/miR-126-5p inhibitor were administered at a dose of 10 nmol. DSF and VX-765 were intravenously administered through the tail vein at a dosage of 50 mg/kg. All mice were treated every other day from day 0.5 and sacrificed on day 11.5 according to previous study [7, 33]. All mice in the treatment groups were sacrificed to evaluate embryonic development, calculate absorption rates, and collect placentas tissue and serum samples.

### Statistical analysis

Statistical analysis work were achieved through GraphPad Prism 10.0, and experimental findings were represented as mean ± standard deviation (mean ± SEM). Paired t-test was employed for analyzing the discrepancy between the two groups, while a one-way ANOVA was applied to evaluate the discrepancy among multiple groups. *P* < 0.05 is considered statistically significant.

## Results

### Caspase-1 is up-regulated in villus tissue of URSA

**Fig. 1:**
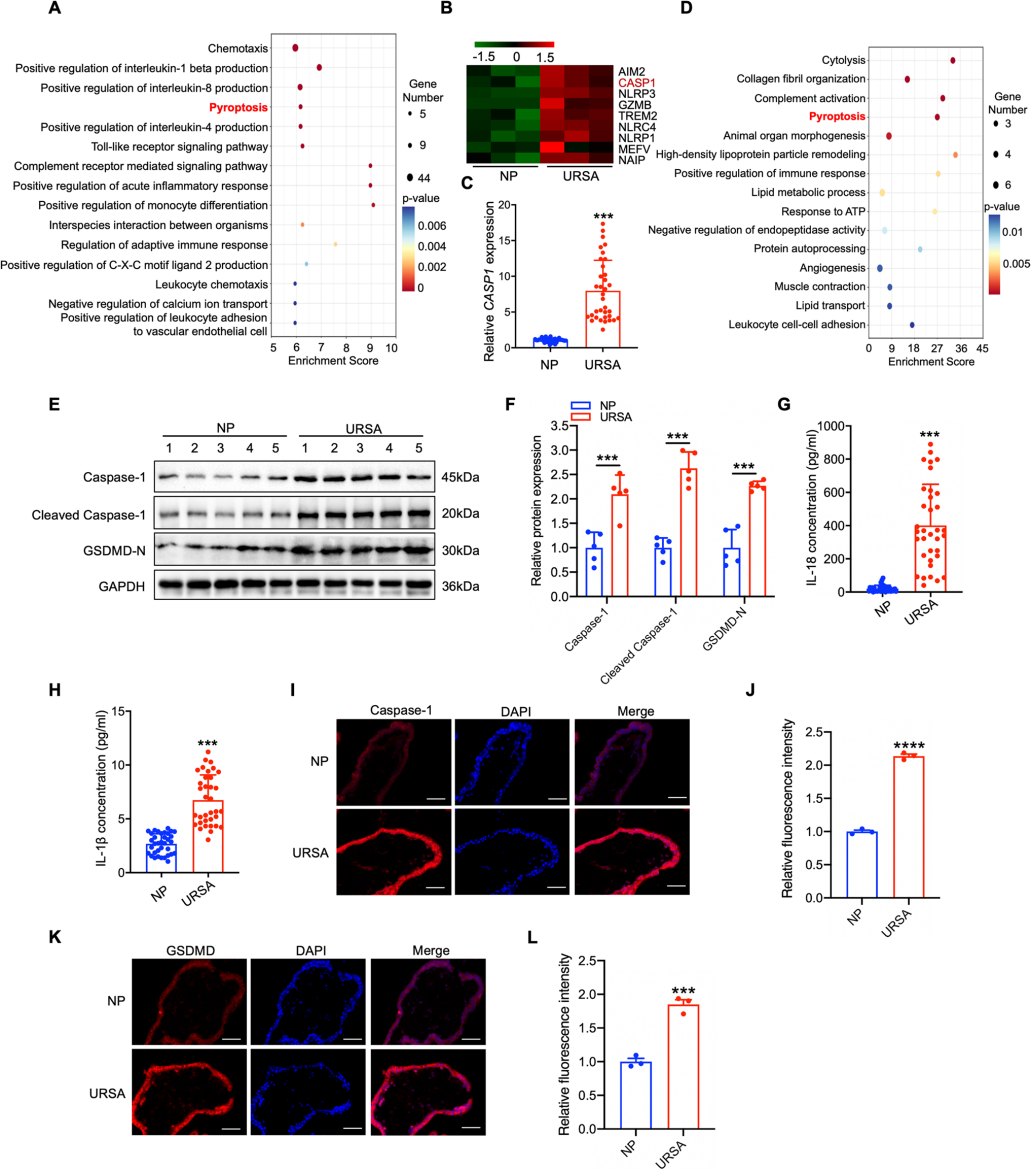
Pyroptosis was significantly activated in the villous tissue of URSA patients**. A** GO pathway enrichment analysis of up-regulated genes in villous tissue of URSA patients. **B** Heatmap showing the up-regulated genes enriched in pyroptosis pathway. **C** The *CASP1* mRNA levels in villous tissue of URSA patients and NP women (n = 35) were determined via qRT-PCR. **D** GO enrichment analysis of up-regulated proteins in villous tissue of URSA. **E** WB revealed the pyroptosis related proteins in villous tissue of URSA patients and NP women (n = 5). **F** The quantitative analyses of pyroptosis related proteins.** G-H** The serum contents of IL-1β/IL-18 in URSA and NP women were analyzed via ELISA (n = 35). **I-J** Immunofluorescence staining of Caspase-1 protein in villous tissue of URSA patients and NP women (Scale bar, 50 µm, 40 ×). **K-L** Immunofluorescence staining of GSDMD protein in villous tissue of URSA patients and NP women (Scale bar, 50 µm, 40 ×). ***P < 0.001, ****P < 0.0001

To explore the biological processes potentially implicated in the pathogenic role of trophoblast cells in URSA, we analyzed the differential expression of genes in villous tissues from URSA patients and NP women using RNA sequencing. Gene Ontology enrichment analysis of up-regulated genes in the villous tissue of URSA patients showed prominent enrichment in the pathway of pyroptosis (Fig. 1A), suggesting that pyroptosis might be activated during the occurrence of URSA. Specifically, we observed a pronounced elevation in *CASP1* mRNA based on the RNA sequencing data (Fig. 1B), and this finding was further validated in an expanded clinical cohort of villous tissues from NP and URSA patients (Fig. 1C). Additionally, 4D label-free quantitative proteomics analysis confirmed the enrichment of the pyroptosis pathway (Fig. 1D). In order to substantiate the process of pyroptosis that was really enhanced and involved in the pathogenesis of URSA, proteins associated with pyroptosis were detected. The result showed significant increases in Caspase-1, cleaved Caspase-1, and GSDMD-N, elucidating a notable activation of pyroptosis in villous of URSA patients (Fig. 1E-F). Moreover, proinflammatory cytokines of IL-1β and IL-18 were found to be greatly elevated in the serum of URSA patients (Fig. 1G-H). Given the complex tissue structure of the villous, we conducted IF staining to anatomically revealed which part of villous primarily contribute to the up-regulation of Caspase-1 and GSDMD. From the results, we concluded that the up-regulation of Caspase-1 and GSDMD mainly occurred in the trophoblast cells of URSA patients (Fig. 1I-L). To sum up, those findings demonstrated that Caspase-1-mediated pyroptosis was increased in trophoblast cells of URSA patients.

### Caspase-1 mediated pyroptosis aggravates the process of miscarriage

**Fig. 2:**
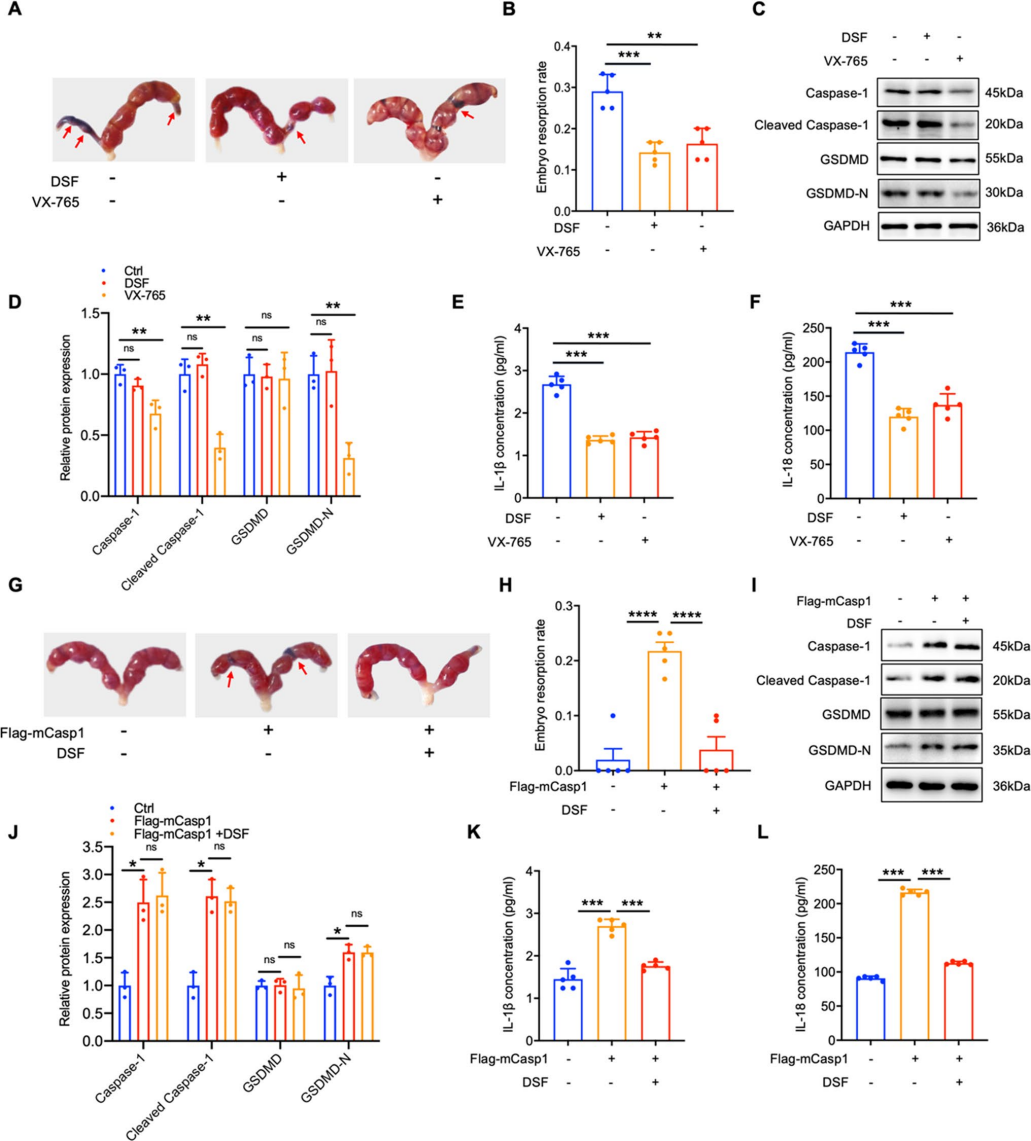
Caspase-1 mediated pyroptosis affects embryo resorption rate. **A** Photograph (red arrow: absorption point) of uterus from URSA pregnant mice (n = 5). **B** The absorption rates were analyzed (n = 5). **C** Proteins related to pyroptosis in the placenta of pregnancy mice were detected by WB (n = 3).** D** The quantitative analyses of pyroptosis related proteins. **E–F** The serum contents of IL-1β/IL-18 of pregnancy mice. **G** Photograph (red arrow: absorption point) of uterus from NP pregnant mice tail vein injected with Flag-mCasp1 or Flag-mCasp1and DSF (n = 5). **H** The absorption rates were analyzed (n = 5). **I** Proteins related to pyroptosis in the placenta of NP pregnancy mice were detected by WB (n = 3).** J** The quantitative analyses of proteins related to pyroptosis. **K-L** The serum contents of IL-1β/IL-18 in serum of pregnancy mice. **P < 0.01, ***P < 0.001, ****P < 0.0001, ns means no statistical difference

To elucidate the effect of pyroptosis driven by Caspase-1 in the development of URSA, we established URSA and NP mouse models. URSA mice were administered the pyroptosis inhibitor DSF and VX-765 (Caspase-1 inhibitor) via the tail vein to examine trophoblast cell pyroptosis and embryo resorption rates. It was observed that the addition of DSF or VX-765 significantly mitigated pregnancy loss in URSA mice (Fig. 2A-B). DSF exhibits strong anti-pyroptosis properties by inhibiting GSDMD-N from drilling holes on the cell membrane, and has no impact on the level of proteins associated with pyroptosis in the placental tissue (Fig. 2C-D), but it reduced IL-1β and IL-18 levels in blood serum of URSA mice (Fig. 2E-F). VX-765 treatment significantly decreased Caspase-1, cleaved Caspase-1, and GSDMD-N in the placental trophoblast, and it also reduced the content of IL-1β and IL-18 (Fig. 2C-F). These results indicated that inhibiting cell pyroptosis can protect from URSA. Additionally, over-expression of mCaspase-1 enhanced the expression of proteins associated with pyroptosis and dramatically aggravated embryo absorption in the placental trophoblast of NP mice (Fig. 2G-J). However, pyroptosis blockade with DSF diminished mCaspase-1 induced pyroptosis and embryo absorption (Fig. 2G-J). Furthermore, in the mCaspase-1 over-expression group, the inflammatory factors IL-1β and IL-18 were significantly up-regulated, while DSF treatment reversed this phenomenon (Fig. 2K-L). Collectively, these data indicated that Caspase-1/GSDMD induced cell pyroptosis was interrelated to the occurrence of URSA.

### Down-regulated miR-126-5p is negatively correlated with Caspase-1 in URSA patients

**Fig. 3:**
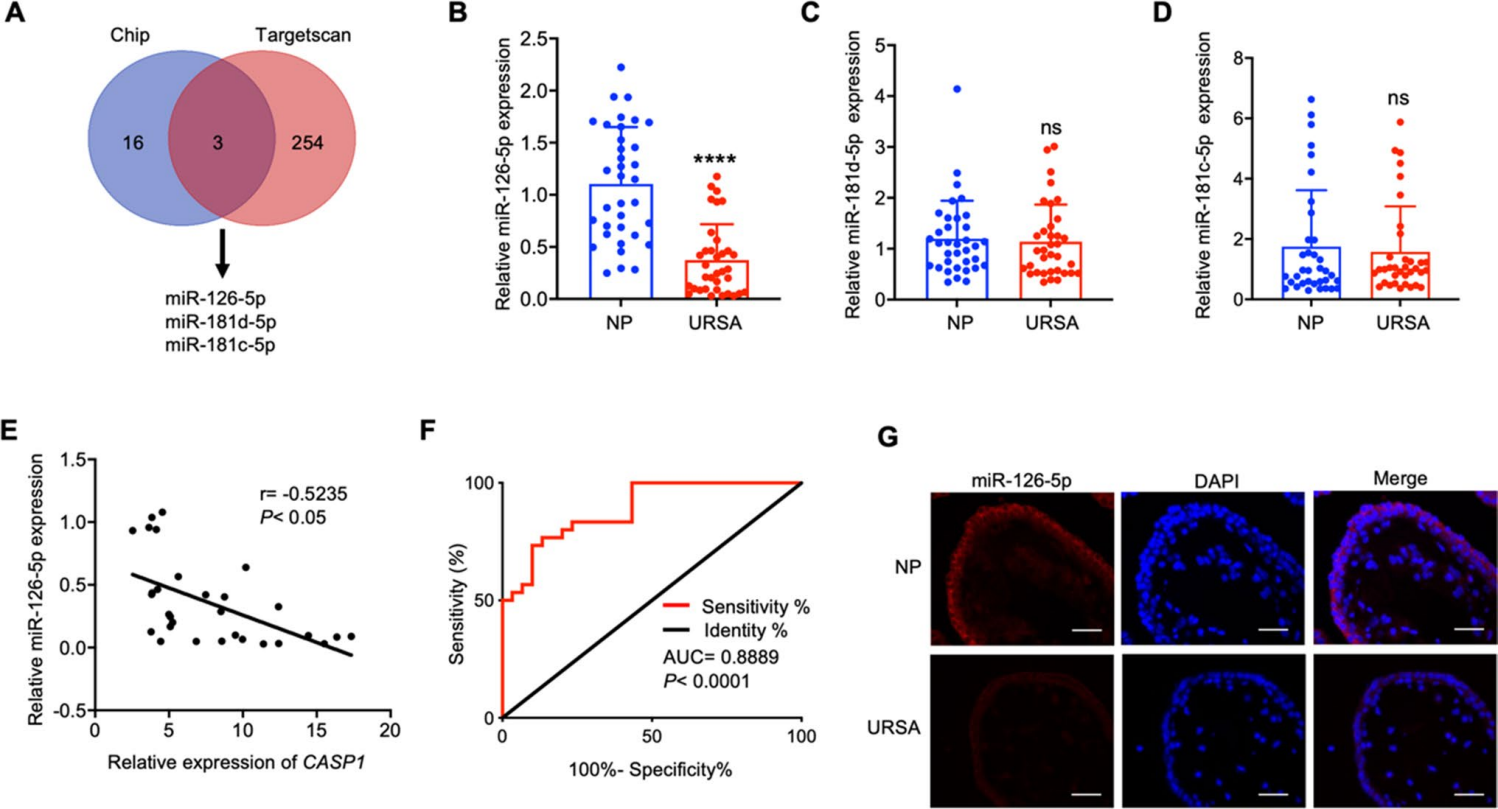
miR-126-5p is down-regulated and negatively correlates with Caspase-1 in URSA patients. **A** Venn diagram displays the 3 down-regulated miRNAs that might complementary combined with *CASP1* analyzed by RNA seq and TargetScan prediction. **B-D** The expression of miR-126-5p, miR-181c-5p and miR-181 d-5p in villous tissues of NP and URSA patients (n = 35) were detected by qRT-PCR. **E** Correlation analysis between *CASP1* and miR-126-5p in villous tissues of URSA patients (n = 30). **F** ROC curve analysis of the diagnostic value of miR-126-5p in villous tissues for URSA (n = 30). **G** Using FISH technique to determine the localization and level of miR-126-5p in villous tissue (scale bar, 50 µm, 40 ×). *** *P* < 0.001, ns means no statistical difference

Given that miRNAs can lead to post transcriptional silencing of Caspase-1, RNA sequencing was conducted on villous tissues from NP and URSA patient. A total of 19 down-regulated miRNAs were detected with at least twofold in URSA villous tissue. Subsequently, TargetScan based analysis highlighted potential miRNAs that target *CASP1*, including miR-126-5p, miR-181c-5p, and miR-181 d-5p (Fig. 3A). Among these candidates, miR-126-5p exhibited the most pronounced down-regulation in the URSA villous tissue, and was selected for further analysis (Fig. 3B-D). Pearson correlation analysis demonstrated a notable inverse correlation between the levels of miR-126-5p and *CASP1* (Fig. 3E). Additionally, miR-126-5p level could effectively discriminated URSA patients from NP women (Fig. 3F). Furthermore, FISH assay revealed that miR-126-5p (red) was predominantly cytoplasmic distributed, and significantly down-regulated in URSA villous tissue (Fig. 3G). Collectively, these discoveries demonstrated that miR-126-5p might contribute to trophoblast cell pyroptosis and the pathogenesis of URSA through its regulation of *CASP1*.

### miR-126-5p alleviates trophoblast pyroptosis by down-regulating Caspase-1

**Fig. 4:**
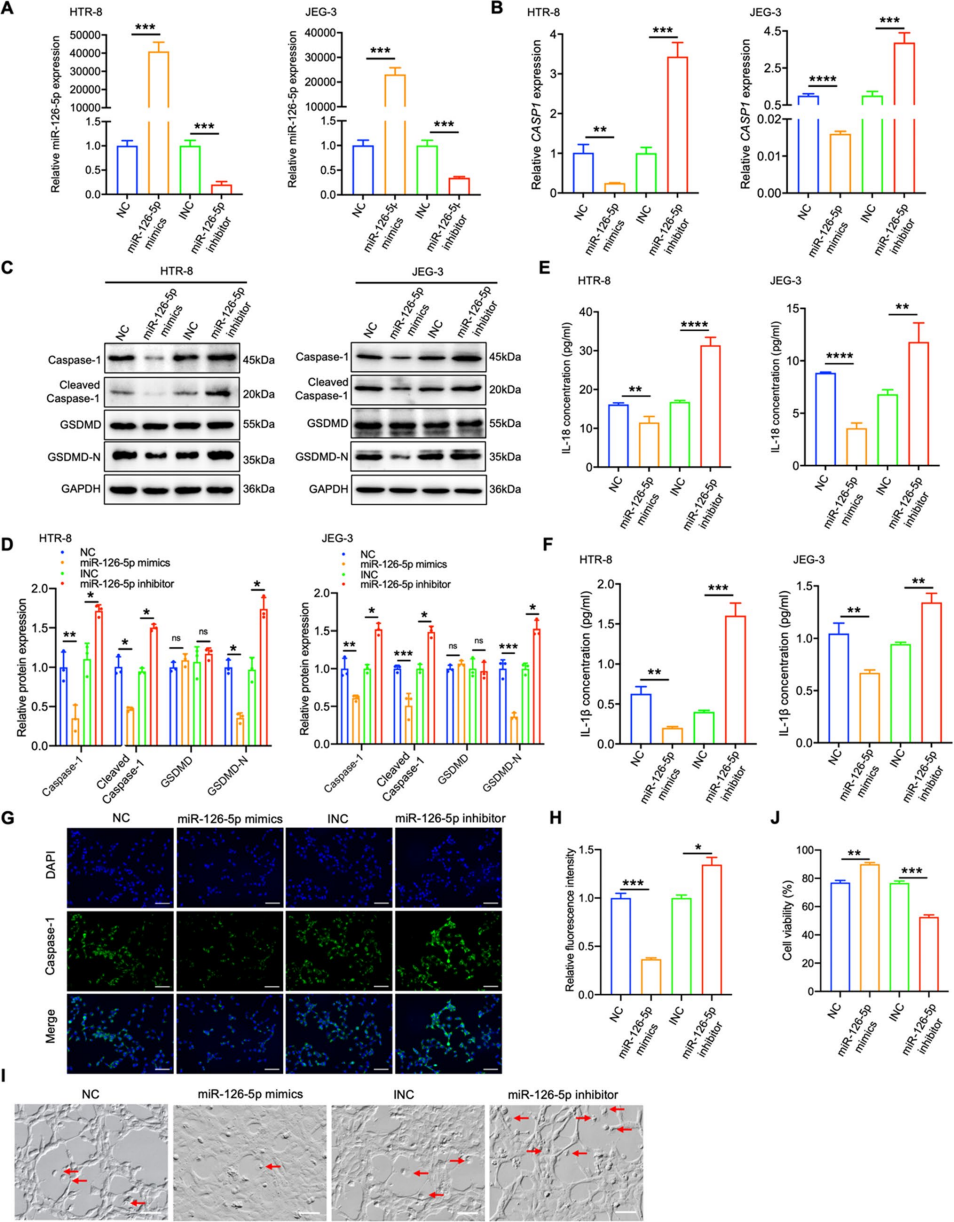
miR-126-5p attenuates trophoblast pyroptosis by suppressing Caspase-1**. A** MiR-126-5p level in HTR-8/SVneo and JEG-3 was detected by qRT-PCR. **B-E** Cells were treated with Hcy following the modulation of miR-126-5p, The *CASP1* mRNA level was analyzed in (**B**), Proteins associated with pyroptosis were detected by WB in (**C**), The quantitative analyses of proteins related to pyroptosis in (**D**), The contents of IL-1β/IL-18 present in the cell supernatant were assessed as shown in (**E–F**). **G-H** IF analyzed Caspase-1 expression in HTR-8/SVneo cells (Scale bar, 50 µm, 20 ×). **I-J** Representative images taken under the microscope of HTR-8/SVneo cells undergoing pyroptosis, arrows indicate the cell with bubbles (Scale bar, 50 µm, 20 ×). *P < 0.05, **P < 0.01, ***P < 0.001, ****P < 0.0001, ns means no statistical difference

To further elucidate the influence of miR-126-5p on trophoblast cell pyroptosis, models for cell pyroptosis were established using HTR-8/SVneo and JEG-3 cells induced by Hcy (Video S1). These cells were treated with miR-126-5p mimics and inhibitor to modulate miR-126-5p level, followed by Hcy treatment to assess its effects on *CASP1* and cell pyroptosis. miR-126-5p mimics effectively elevated miR-126-5p expression, while the inhibitor suppressed it (Fig. 4A). Elevated miR-126-5p expression markedly reduced both *CASP1* mRNA levels and Caspase-1/GSDMD-N protein levels, whereas miR-126-5p inhibition reversed these suppressive effects (Fig. 4B-D). Moreover, administration of miR-126-5p mimics or inhibitors resulted in a significant reduction or elevation of IL-1β and IL-18 concentrations in the culture medium, respectively (Fig. 4E-F). IF experiments also validated that over-expression of miR-126-5p can inhibit Caspase-1 expression, while miR-126-5p suppression displayed the opposite effects (Fig. 4G-H). Consistent with these molecular alterations, morphological analysis of HTR-8/SVneo trophoblasts revealed that miR-126-5p mimics attenuated characteristic pyroptotic features, including membrane blebbing and cytoplasmic swelling. Conversely, miR-126-5p inhibition exacerbated these pyroptosis-associated morphological changes (Fig. 4I-J).

**Fig. 5:**
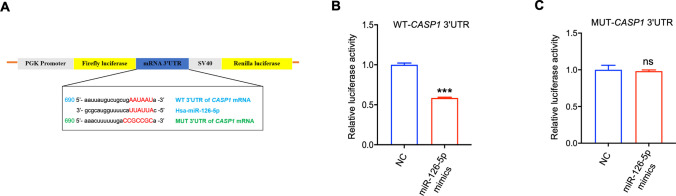
CASP1 is a direct target of miR-126-5p.** A** The schematic diagram shown the binding sequence (WT) and mutation sequence (MUT) of miR-126-5p to *CASP1* 3'UTR. **B-C** The luciferase activity was determined in 293 T cells. ***P < 0.001, ns means no statistical difference

Subsequently, the putative binding sites between miR-126-5p and the *CASP1* 3'UTR were predicted using TargetScan 7.2 (Fig. 5A). Dual-luciferase reporter analysis revealed that the transfection of miR-126-5p mimics caused a marked decrease in luciferase reporter activity of WT 3'UTR, whereas no notable changes were observed in the mutant (MUT) 3'UTR (Fig. 5B-C). These findings demonstrated that miR-126-5p negatively regulate *CASP1* level by targeting its 3'UTR, thereby influencing trophoblast cell pyroptosis and the release of associated inflammatory factors.

### miR-126-5p reduces embryo resorption rate by down-regulating Caspase-1-dependent pyroptosis

**Fig. 6:**
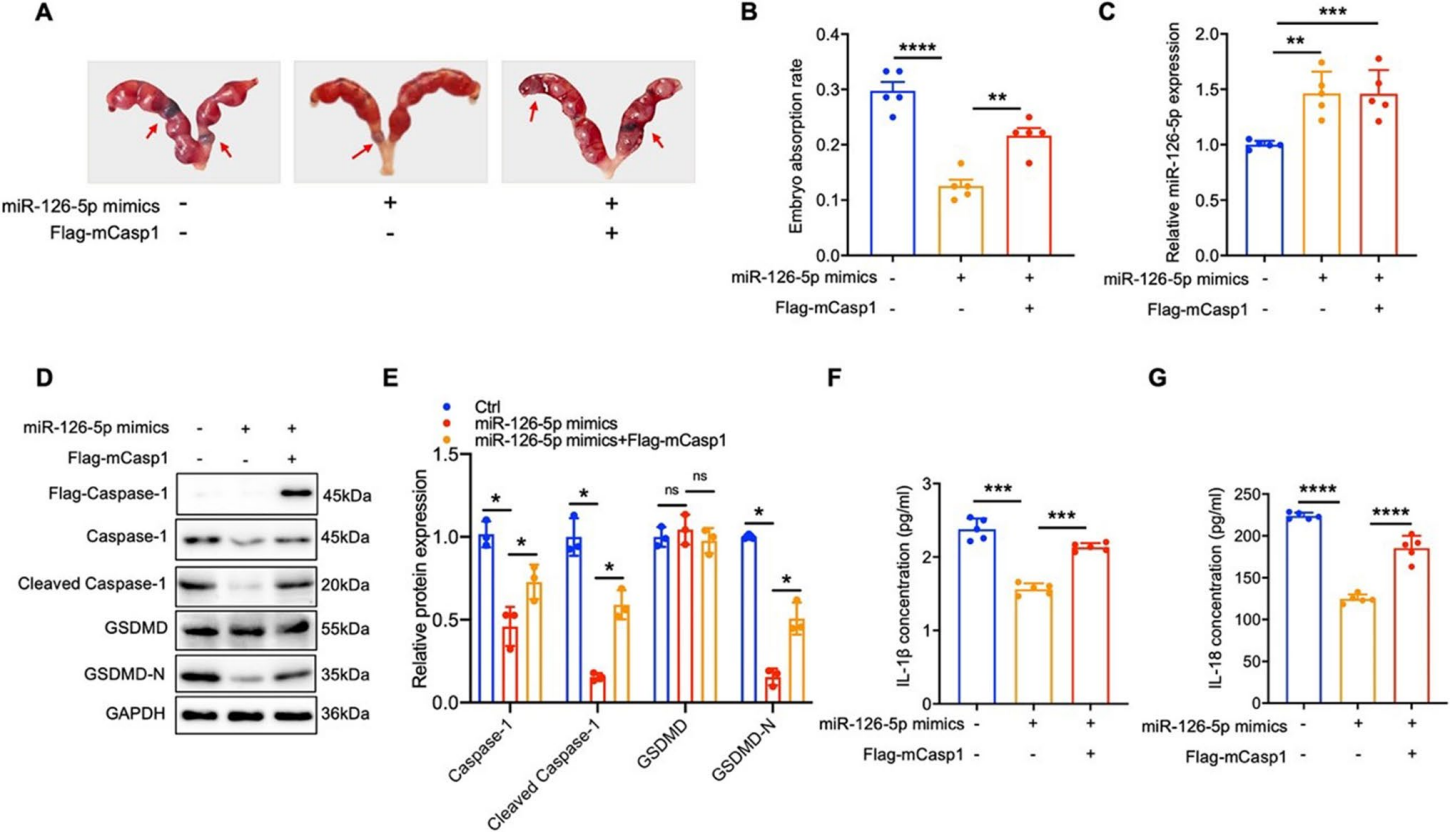
miR-126-5p reduces embryo resorption rate by down-regulating Caspase-1 mediated pyroptosis in URSA mice. **A-B** The embryo resorption rates were measured across three groups of URSA mice, with arrows highlighting instances of embryo resorption (n = 5). C The miR-126-5p levels in the placental tissue of pregnant mice. **D** WB analysis was performed to assess the expression of pyroptosis-related proteins in the placental tissue of pregnant mice (n = 3). **E** The quantitative analyses of proteins related to pyroptosis. **F-G** Serum levels of IL-1β and IL-18 in pregnant mice. *P < 0.05, **P < 0.01, ***P < 0.001, ****P < 0.0001, ns means no statistical difference

**Fig. 7:**
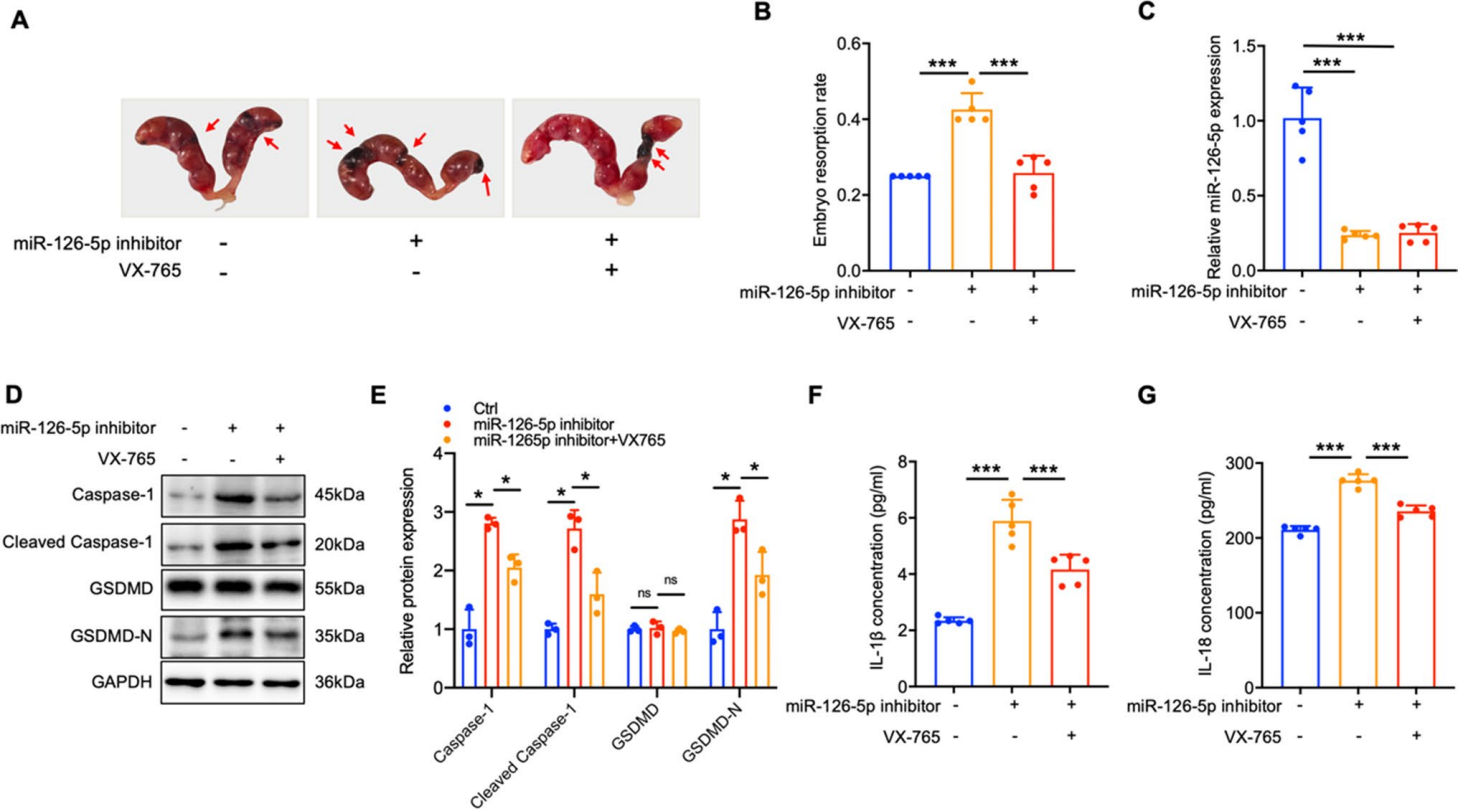
Inhibition of miR-126-5p aggravate embryo loss by up-regulating Caspase-1 mediated pyroptosis**. A-B** The embryo resorption rates were measured across three groups of URSA mice, with arrows highlighting instances of embryo resorption (n = 5). **C** The expression levels of miR-126-5p in the placental tissue of pregnant mice. **D** The expression levels of pyroptosis-related proteins in the placental tissue of pregnant mice were analyzed by WB (n = 3). **E** The quantitative analyses of proteins related to pyroptosis. **F-G** The serum level of IL-1β/IL-18 in pregnancy mice. *P < 0.05, ***P < 0.001

To further define miR-126-5p’s involvement in the occurrence of URSA by regulating Caspase-1-mediated trophoblast pyroptosis, we simultaneously injected miR-126-5p mimics and mCaspase-1 into the tail vein of pregnant URSA mice to visualize the effect of miR-126-5p on mCaspase-1 induced pregnancy loss. Meanwhile, we also observed the inhibition of pyroptosis on miR-126-5p inhibitor induced embryo absorption. Our results demonstrated that administration of miR-126-5p mimics effectively improved its content in the placenta, and prominently diminished embryo resorption rate in URSA mice, however, mCaspase-1 over-expression reversed the therapeutic effects induced by miR-126-5p (Fig. 6A-C). Mechanistically, up-regulation of miR-126-5p inhibited Caspase-1 expression, which in turn ameliorated the production of cleaved Caspase-1 and GSDMD-N in placental tissue, and the secretion of IL-1β and IL-18 (Fig. 6D-G). Conversely, over-expression of mCaspase-1 by Flag-mCasp1 reversed the reductions caused by miR-126-5p (Fig. 6D-G). Besides, miR-126-5p inhibitor effectively diminished its content in the placenta, and notably improved embryo resorption rates in URSA mice, which could be attenuated by VX-765 (Fig. 7A-C). Notably, inhibition of miR-126-5p significantly elevated the expression of Caspase-1, cleaved Caspase-1, and GSDMD-N proteins in placental tissue, as well as serum levels of IL-1β and IL-18, while VX-765 treatment effectively reversed these effects (Fig. 7D-G). Collectively, these data elucidated that miR-126-5p suppresses trophoblast pyroptosis by targeting *CASP1*, and holds the potential as the therapeutic target for URSA patients.

## Discussion

The fetus, akin to an allogeneic transplant, can develop normally within the mother without rejection, relying on immune tolerance at the maternal fetal interface [34]. Numerous studies have showed that the occurrence of URSA is associated with immune disorders and inflammation at this interface [28, 35, 36]. Recent studies identify pyroptosis as a contributor to excessive or sustained inflammation, which will eventually lead to RSA [28, 35, 36]. Additionally, exposure to environmental toxins like BaP or BPDE induces trophoblast cell pyroptosis and is associated with RSA occurrence [37]. These studies underscore the pivotal role of pyroptosis in RSA. Our resarch found that the alteration of Caspase-1 and the Caspase-1-mediated cell pyroptosis were dramatically increased in the villous tissue of URSA patients. Concurrently, miR-126-5p content noticeably decreased in URSA patients and negatively correlated with CASP1 mRNA. More importantly, we provided evidences that reduction of miR-126-5p facilitated trophoblast pyroptosis by disinhibiting Caspase-1/GSDMD pathway. MiR-126-5p inhibits Caspase-1 expression by binding CASP1 mRNA 3'UTR. Furthermore, our findings demonstrated that miR-126-5p over-expression suppressed trophoblast pyroptosis, thereby reducing embryo resorption rates in vivo.

Pyroptosis, a novel recognized form of inflammatory programmed cell death, differs from other form of cell death in its mechanism. In pyroptosis, activated Caspases cleave GSDM family proteins, resulting in the release of pore-forming GSDM proteins that oligomerizes to create pores on the cell membrane. This process leads to ion influx, cell swelling, rapid membrane rupture, and further amplifies the inflammatory response through the release of inflammatory cytokines such as IL-1β and IL-18 [38, 39]. Appropriate pyroptosis is one of the main means for the body's immune system to combat pathogen invasion, however, excessive pyroptosis can lead to pathological reactions [18–20, 40]. Research on RSA highlights improved levels of high mobility group box 1 (HMGB1) in decidual tissue, significantly promoting cell pyroptosis and inflammatory factor release through Caspase-1 activation [41]. Studies also linked benzo(a)pyrene-7,8-dihydrodiol-9,10-epoxide, a persistent organic pollutant and endocrine disruptor, with trophoblast cell pyroptosis induction and URSA [37]. These studies implied that pyroptosis play a crucial role at maternal–fetal interface in RSA. In our study, RNA sequencing and 4D label-free quantitative proteomics revealed significant up-regulation of pyroptosis in villous tissue of URSA patients. Concurrently, proteins associated with pyroptosis, including Caspase-1, cleaved Caspase-1, GSDMD-N in villous tissue, along with proinflammatory cytokines of IL-1β and IL-18 in serum, were all markedly elevated in URSA patients. Moreover, Caspase-1 inhibitor significantly reduced the embryo absorption rates, placental levels of pyroptosis-related proteins, along with the serum content of IL-1β and IL-18 in URSA mice. Conversely, Caspase-1 over-expression showed opposite results. These findings strongly implicate Caspase-1-mediated pyroptosis in the pathogenesis of URSA.

MiRNAs are a class of pervasive and conserved non-coding RNAs, that function as negative regulators by binding to target genes'3'UTR [42]. Extensive evidence links miRNA regulation of cell pyroptosis to the occurrence of various diseases [7, 43]. For instance, down-regulation of miR-513c-5p in the peripheral blood mononuclear cells accelerates pyroptosis by up-regulating Caspase-1 and contribute to the pathogenesis of deep vein thrombosis (DVT) [44]. Furthermore, miR-223-3p in extracellular vesicles of human medulla ossium mesenchymal stem cells alleviate inflammation and pyroptosis in acute kidney injury (AKI) by targeting HDAC2 and enhancing SNRK transcription [45]. Given miRNAs'significant role in RSA [46, 47]. We hypothesized that miRNA-regulated pyroptosis contributes to URSA development. In this work, miR-126-5p was suppressed in the villous tissue of URSA patients, which can distinguish URSA patients from controls with high sensitivity.

miR-126-5p is located in the 9q34.3 region of human chromosome. It has been extensively studied for its significant roles in various tumors, such as renal cell carcinoma, colorectal cancer and ovarian cancer [48–51]. In addition, miR-126-5p down-regulation contributes to atherosclerosis by reducing endothelial cell proliferation at the lesion sites [52]. Conversely, up-regulated miR-126-5p promotes inflammatory factors release, increasing vascular disease risk in diabetes [53]. It can also mitigate sustained inflammatory factor release in HIV-1 infected monocytes, highlighting its crucial role in inflammatory response [54]. Our findings demonstrated that miR-126-5p over-expression reduces Caspase-1 levels by directly interacting with the 3'UTR of *CASP1* mRNA, thereby decreasing trophoblast pyroptosis and IL-1β and IL-18 secretion. Conversely, miR-126-5p knockdown showed the opposite results. In vivo supplementation of miR-126-5p markedly reduces embryo resorption rates and placental pyroptosis levels in URSA mice, while miR-126-5p inhibition showed the opposite effects. Moreover, Flag-mCasp1 over-expression in vivo was able to reverse miR-126-5p mimics-induced decreases in embryo resorption rate and pyroptosis level.

**Fig. 8:**
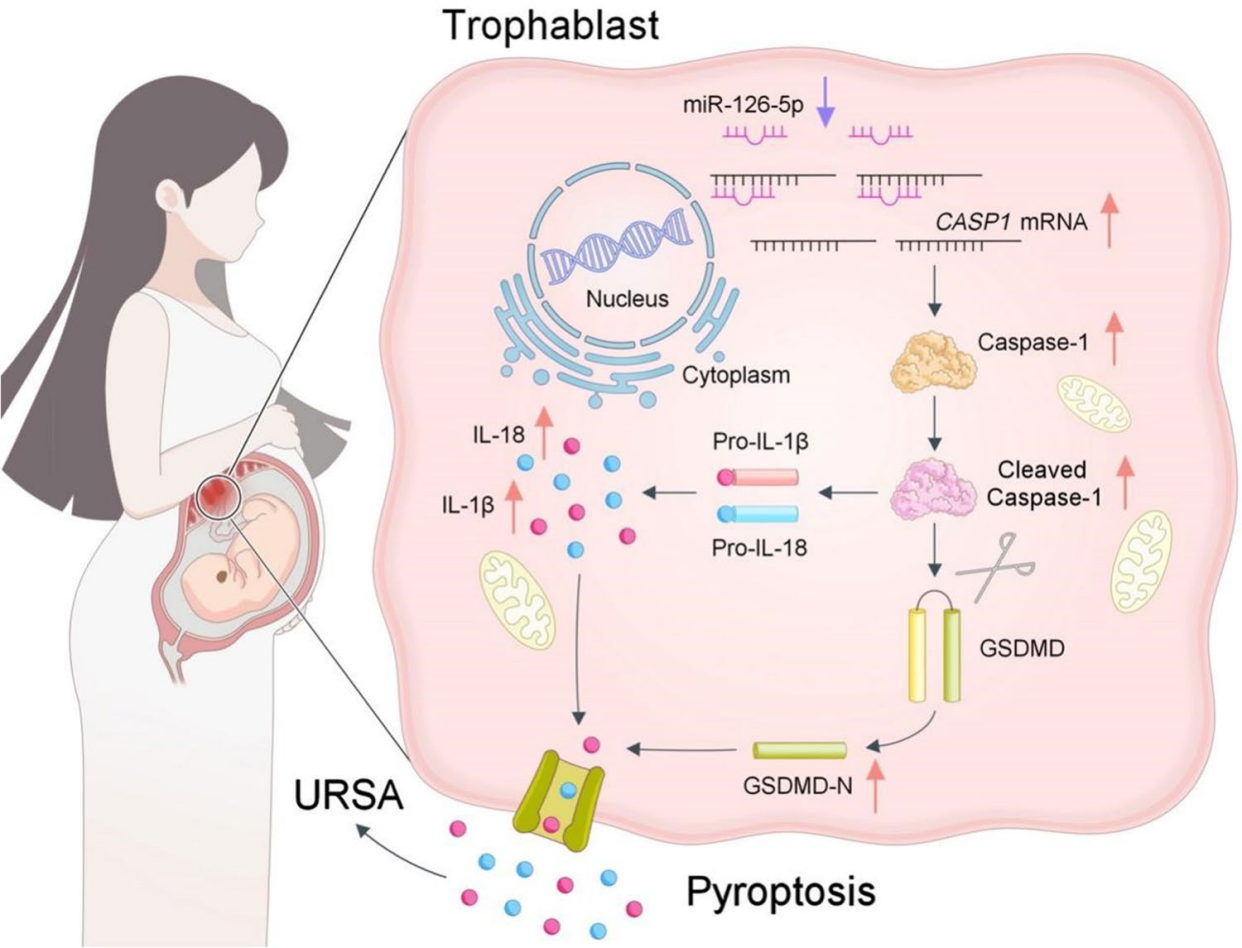
Schematic diagram of miR-126-5p mediated trophoblast pyroptosis in URSA. Downregulation of miR-126-5p in trophoblasts attenuates its inhibitory effect on Caspase-1 expression, thereby promoting trophoblast pyroptosis-mediated embryo loss and contributing to the pathogenesis of URSA

Overall, this study highlights the crucial role of miR-126-5p in Caspase-1 mediated trophoblast pyroptosis. miR-126-5p exerts its inhibitory effects on trophoblast pyroptosis by targeting the 3'UTR of *CASP1* mRNA, thereby suppressing the Caspase-1/GSDMD signaling pathway. These data revealed that reduced content of miR-126-5p in the villous tissue of URSA patients lead to up-regulation of Caspase-1 and activation of Caspase-1 mediated trophoblast pyroptosis, resulting in excessive or sustained inflammation at the maternal fetal interface, and finally contributing to the occurrence of URSA (Fig. 8). Additionally, it is noteworthy atypical pyroptosis driven by Caspase-4/5/11 may also play a role in URSA pathogenesis. Further research is warranted to elucidate the regulatory function of miR-126-5p on these atypical pathways.

## Supplementary Information

Below is the link to the electronic supplementary material.

Supplementary file1 (MP4 24080 KB)

## Data Availability

Any data reported in this paper is available from the corresponding author upon reasonable request.
